# Fluctuating obliterative bronchiolitis in RET-mutant medullary thyroid cancer patient treated with selpercatinib

**DOI:** 10.1530/ETJ-24-0189

**Published:** 2024-09-19

**Authors:** Carla Gambale, Alessandro Prete, Chiara Romei, Alessandro Celi, Rossella Elisei, Antonio Matrone

**Affiliations:** 1 Department of Clinical and Experimental Medicine, Unit of Endocrinology, Pisa University Hospital, Pisa, Italy; 2 Department of Diagnostic Imaging, Unit of Radiology, Pisa University Hospital, Pisa, Italy; 3 Department of Surgery, Medicine, Molecular Biology and Critical Care, Respiratory Pathophysiology Unit, Pisa University Hospital, Pisa, Italy

**Keywords:** adverse events, highly selective RET inhibitor, medullary thyroid cancer, obliterative bronchiolitis, selpercatinib

## Abstract

Highly selective RET inhibitor selpercatinib has demonstrated notable efficacy in advanced/progressive RET-mutant medullary thyroid cancer (MTC) patients. However, despite a more tolerable toxicity profile than multikinase inhibitors, peculiar adverse events (AEs) have been described. Obliterative bronchiolitis (OB) is a respiratory disease characterized by inflammation and fibrosis in small conducting airways. We evaluated a 70-year-old man with advanced RET-mutant MTC who developed OB during treatment with selpercatinib. Radiological features of OB occurred early and persisted during selpercatinib treatment, with a waxing and waning pattern. Notably, a partial response of MTC was achieved during the treatment, and selpercatinib was never reduced or interrupted. The almost complete absence of symptoms and the fluctuating trend, without specific treatment for OB, suggested that it is necessary to carefully evaluate the risks mediated by this AE with the risks of modifying or discontinuing the anti-cancer therapy.

**Established facts**

- Selpercatinib, a highly selective RET inhibitor, has demonstrated notable efficacy in advanced/progressive RET-mutant medullary thyroid cancer patients.
- Despite a more tolerable toxicity profile than multikinase inhibitors, atypical adverse events have been described.

**Novel insights**

- We present the first reported case of obliterative bronchiolitis occurring during treatment with selpercatinib in an advanced RET-mutant medullary thyroid cancer patients.

## Introduction

Medullary thyroid cancer (MTC) originates from thyroid C cells producing calcitonin (CTN) and is classified as a neuroendocrine tumor. MTC can be sporadic or hereditary, with the rearranged during transfection proto-oncogene (*RET*) playing a pivotal role in tumorigenesis in both scenarios (1). Almost all hereditary cases carry germline *RET* mutations. Conversely, somatic *RET* mutations are present in about 50% of all sporadic cases and even more, up to 85%, if they are advanced/metastatic (1).

According to European Medicines Agency (EMA) indications, two multikinase inhibitors (MKIs) (i.e. vandetanib and cabozantinib) and one highly selective* RET* inhibitor (i.e. selpercatinib) have been approved for the treatment of progressive advanced/metastatic MTC (2). Due to their nature, vandetanib and cabozantinib inhibit a broad spectrum of receptors, including the RET receptor, although with a lower affinity compared to the others. Conversely, the activity of selpercatinib against *RET* is very high and quite specific (3).

Efficacy and toxicity of selpercatinib compared to the standard of care (i.e. cabozantinib or vandetanib) for the treatment of advanced/metastatic progressive *RET*-mutant MTC, naive to any prior MKIs therapy, have been recently investigated in the phase III clinical trial LIBRETTO-531 (ClinicalTrials.gov no. NCT04211337) (4). In LIBRETTO-531, selpercatinib showed higher progression- and treatment failure-free survival compared with cabozantinib or vandetanib. Calcitonin (Ct) and CEA values showed a significant decrease from the first days after starting treatment. However, cases of increased CEA values, despite the objective tumor response to selpercatinib, have been described (5). Notably, significant differences were observed in dose reduction and definitive treatment discontinuation due to adverse events (AEs), being 38.9% and 4.7% in the selpercatinib group and 77.3% and 26.8% in the cabozantinib/vandetanib group. These data confirmed the superior efficacy and the less severe toxicity profile of selpercatinib compared to previous treatments. Although several AEs were shared in both groups (i.e., hypertension, increased transaminase, fatigue, etc.), other distinctive AEs of selpercatinib therapy, such as chylous effusions, gastrointestinal adverse events, and erectile dysfunction, have been recently reported (6, 7, 8, 9). We describe here a new possible and peculiar AE observed during treatment with selpercatinib and we report also how we managed it, thus avoiding interruptions and discontinuation.

## Case presentation

We describe the case of a 70-year-old man who came to our attention in May 2019 due to the occasional finding of a large thyroid nodule in the right lobe. The medical history of the patient showed a type 2 diabetes well controlled under specific therapy and benign prostatic hypertrophy. He was an ex-smoker (5 cigarettes/day for 2 years, stopped in 1990). A neck ultrasound (US) performed in our department confirmed the presence of a large (8 cm) right thyroid nodule with multiple lymph nodes suspicious for metastases in the right latero-cervical compartment. Fine needle aspiration cytology of both the nodule and one of the latero-cervical lymph nodes was suspicious for MTC and pre-operative CTN values were 26,700 ng/L (normal values <18 ng/L). Due to the high values of CTN, a total body computed tomography (CT) scan with i.v. contrast medium was performed, but no suspicious distant metastases were highlighted. The patient was treated with total thyroidectomy, and central and right latero-cervical lymph node dissection in June 2019. Histology confirmed an MTC (8 cm), with minimal extra-thyroidal extension and vascular invasion but with <4 neoplastic emboli and multiple lymph node metastases in the central and right latero-cervical compartments (pT3aN1bMx, according to TNM 8th edition) (10). No germline *RET* mutations were found, but a somatic* RET* Met918Thr was found in the tumoral tissue. At the first post-operative evaluation (3 months after surgery), high CTN values (1,717 ng/L) and small suspicious metastatic right lateral cervical lymph nodes at the neck US persisted. A total body CT scan with i.v. contrast medium was performed but again no distant metastases were detected. Therefore, an active surveillance strategy was chosen. At the following evaluation (June 2020) CTN values increased (3254 ng/L) and 2 small (<1 cm) suspicious liver metastases were detected by abdomen magnetic resonance imaging (MRI). Because of the relatively small dimensions of the metastases, the multidisciplinary team decided to continue with the active surveillance strategy with clinical, biochemical, and imaging examinations every 6 months. The disease remained stable up to April 2021 when a CT scan showed an increase in both lymph nodes and liver metastases dimensions and, according to RECIST 1.1, the disease was considered progressive. Interestingly, no suspicious lung lesions for infective/inflammatory disease were described in this CT scan. CTN and carcinoembryonic antigen (CEA) values were 4280 ng/L and 40 µg/L, respectively. The patient started systemic treatment with selpercatinib in April 2021 at the initial dose of 160 mg/BID. CTN and CEA values dropped to 208 ng/L and 39.5 µg/L after one week and to 43 ng/L and 30.7 µg/L after 4 weeks of treatment, respectively. Eight weeks after the beginning of the selpercatinib therapy, the first imaging evaluation (i.e. total body CT scan with i.v. contrast and liver MRI) showed a partial response of the tumor according to RECIST 1.1. Of note, at this first CT scan evaluation, the radiologist described the presence of a few centrilobular micronodules at the posterior segment of the right lung superior lobe, but the patient did not report any symptoms.

### Diagnostic assessment, treatment, and follow-up

**Figure 1:**
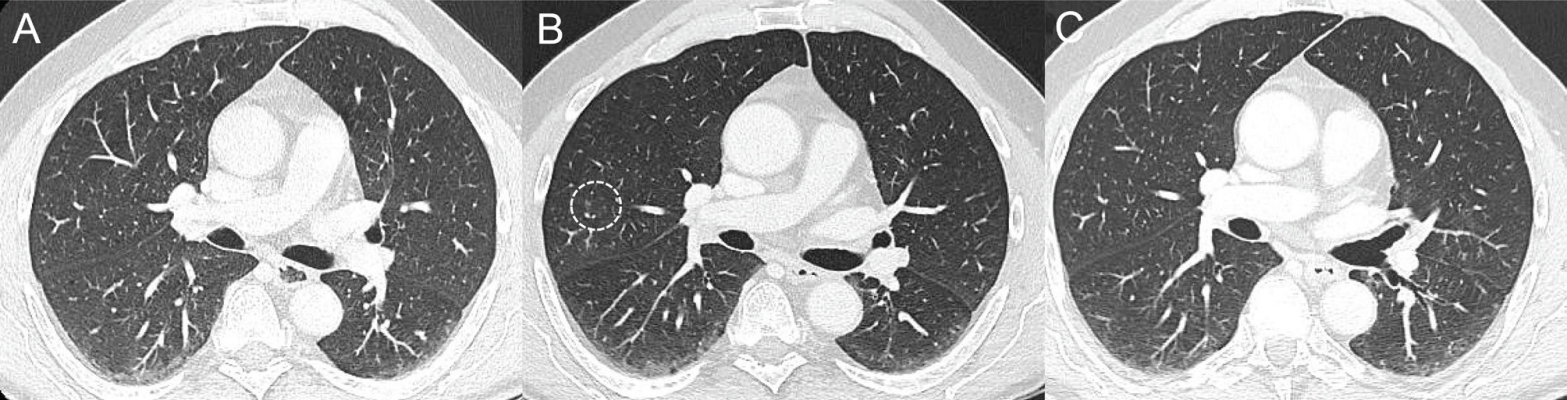
Three CT scans performed before and a few months after starting selpercatinib. (A) negative lung CT of the patient in April 2021, before starting selpercatinib. (B) evidence of a few centrilobular micronodules in the upper right lung lobe (white circle) in a CT scan of June 2021. (C) CT scan of August 2021 showing the complete resolution of the micronodules.

This peculiar aspect of the lung, which was absent on the CT scan before starting selpercatinib (Fig. 1A), appeared early during the first imaging evaluation 8 weeks after the beginning of treatment (Fig. 1B). Since no related symptoms were reported by the patient or observed during the clinical visit, we decided not to perform any specific treatment (i.e. antibiotics, corticosteroids) and to closely monitor the clinical condition of the patient and the aspect of the lung on the CT scan, pursuing the scheduled imaging for the control of MTC. The following CT scan of August 2021 showed the spontaneous and complete resolution of the lung abnormalities (Fig. 1C), while in October 2021, some centrilobular micronodules at the apical segment of the right upper lung lobe reappeared, although the patient remained asymptomatic. In November 2021, the patient reported the onset of a wet cough with phlegm (Common Terminology Criteria for Adverse Events (CTCAE) - G2) (11), for which, suspecting an airway infection, we decided to start empiric treatment with amoxicillin/clavulanic acid (875 mg/125 mg BID for 6 days).

**Figure 2:**
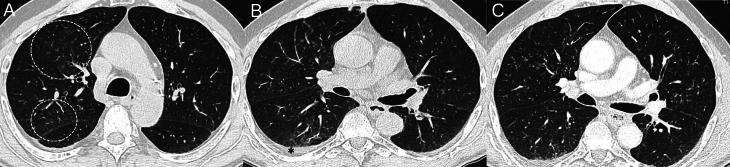
Three different CT scans showing relevant lung involvement. (A) in July 2022, the CT scan showed a diffuse ground-glass area with centrilobular micronodules and bronchiolar wall thickening in the right upper lung lobe (white circles). (B) in December 2022, the CT scan demonstrated the appearance of a new ground-glass area with bronchiolar wall thickening in the right lung with pleural effusion, as indicated by the asterisk (*). 0(C) in February 2024, the last CT scan showed multiple centrilobular micronodules with bronchiolar wall thickening diffuse in both lungs.

The cough resolved after the treatment, and a new CT scan was scheduled for January 2022. Unfortunately, the patient contracted a SARS-Cov-2 infection, without relevant symptoms, and for this reason, the CT scan was performed in February 2022 after the resolution of the SARS-Cov-2 infection without any treatment. At this point, the CT scan did not show the micronodules anymore. In April 2022, the CT scan unexpectedly showed the reappearance of the centrilobular micronodules at the anterior segment of the right upper lung lobe, and the patient suffered cough episodes (CTCAE G2) again, for which empirical treatment with amoxicillin/clavulanic acid was started again with the same modality and with the resolution of symptoms after 7 days. Despite the absence of symptoms, in July 2022, there was a relevant worsening of the lung aspect at the CT scan, characterized by the presence of a diffuse ground-glass area with centrilobular micronodules and bronchiolar wall thickening in the right upper lobe associated with the appearance of pleural effusion (CTCAE G1) (Fig. 2A). Considering the absence of symptoms, we decided to continue selpercatinib treatment at the initial dose of 160 mg/BID and not to not perform any specific treatment. A spontaneous improvement of lung appearance and pleural effusion was observed on the following CT scan (September 2022), but a further worsening in the absence of specific symptoms was observed in December 2022 (Fig. 2B). In February 2023, the cough reappeared (CTCAE G1), no therapy was performed, and the cough spontaneously resolved, but the following CT scan performed about one month later showed a new improvement in the imaging characterized by a more nuanced appearance of the lesions. The following CT scans (March and May 2023) confirmed a spontaneous, quite complete resolution of the lung condition, with a very slight worsening in August 2023. At this point, a consultation with the radiologist and the pulmonologist was performed, and both agreed that the clinical symptoms (i.e. intermittent cough) and the CT features (i.e. bronchiolar wall thickening, diffuse ground-glass area, and centrilobular micronodules) were highly suggestive of obliterative bronchiolitis (OB). However, no specific therapies were suggested, and selpercatinib treatment was continued at the same dose (160 mg/BID) for the duration of the follow-up. Unexpectedly, the last CT scan in February 2024 showed a relevant worsening of the lung aspect with multiple centrilobular micronodules with bronchiolar wall thickening diffuse in both lungs (Fig. 2C). Spirometry was performed, and, since no significant alterations were highlighted, no specific therapy was started, but the surveillance was confirmed. At the same time, the tumoral disease was stable after the initial experience of a partial response, and at the data lock (February 2024), the patient is still in good clinical condition and on selpercatinib treatment at the same daily dose of 160 mg/BID.

**Figure 3:**
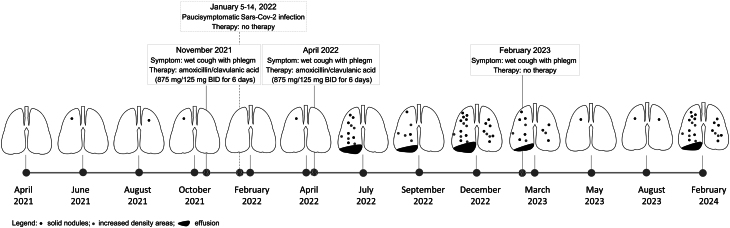
Timeline of the lung involvement, symptoms experienced, and therapies used, according to the periodic CT scan imaging during the follow-up.

During the treatment with selpercatinib, the patient developed other AEs mainly of mild (CTCAE G1) (i.e. fatigue, increase in creatinine, pleural effusion, ascites) or moderate (CTCAE G2) (i.e. arterial hypertension, decrease in lymphocytes) grade and managed according to the standard of care without any need to interrupt or discontinue the therapy. A summary timeline of the lung involvement, correlated symptoms, and therapies performed according to CT scan findings is represented in Fig. 3.

## Discussion

The highly selective *RET* inhibitors (e.g. selpercatinib and pralsetinib) have led to a paradigm change in the treatment of *RET-*mutated thyroid cancers, thanks to their strong efficacy and tolerable toxicity profile. However, beyond AEs shared with MKIs, some AEs seem to be peculiar to highly selective *RET* inhibitors (6, 7, 8). Having a complete landscape of those peculiar AEs is crucial to improving their prevention and management, mainly aiming to prolong anti-cancer treatment as long as possible. Here, we are presenting the first case of suspicious OB during selpercatinib treatment.

Pulmonary toxicities are often described during MKI treatment (12). In particular, they encompass pneumonitis, interstitial lung disease, effusions, thromboembolic disease, tumor cavitation, and, although very rarely, also OB (13). These reports usually include patients with pulmonary tissue impaired by primary cancer or metastasis. However, in these cases, pulmonary toxicity could be due to MKIs’ peculiar mechanism of action (i.e. epidermal growth factor receptor inhibition on type II pneumocytes) (14), but also directly due to cancer (i.e. paraneoplastic syndrome) (15). In the present case, the patient developed pulmonary toxicity during selpercatinib treatment in the absence of smoking habits, lung cancer, or MTC lung metastasis, thus representing a neat model of pulmonary toxicity.

Pulmonary toxicities have been rarely reported during selpercatinib treatment (16). However, in the LIBRETTO-001 trial (ClinicalTrials.gov no. NCT03157128), evaluating toxicity and efficacy of selpercatinib in patients affected by *RET-*mutated MTC with and without previous vandetanib or cabozantinib treatment, cough and dyspnea were reported in 18% and 16%, respectively (17). Moreover, in September 2022, interstitial lung disease (ILD)/pneumonitis was added to the ‘highlights of prescribing information’ by the FDA (18). In this view, pulmonary toxicity potentially linked to selpercatinib treatment could be more heterogeneous than conceived in the past, including also pulmonary AEs often observed with other MKIs.

OB is characterized by inflammation and fibrosis in conducting airways with a diameter of less than 2 mm (19). Mechanistically, both these phenomena could be triggered by an injury occurring to small airways, which promotes excessive fibroproliferation, inflammation, and aberrant tissue repair (20). Many toxic fumes have been associated with OB, such as fiberglass, sulfur mustard, and nitrogen oxides (20). Moreover, the association of OB with graft-versus-host disease (GVHD) raised the hypothesis of immune system involvement, and both T-cell- and antibody-mediated systems involvement has been reported (21, 22). Interestingly, the *RET* gene has been found expressed in fibroblasts and immune cells (16, 23). Moreover, *RET* inhibition has been associated with immune-mediated hypersensitivity, particularly in cases previously treated with immune checkpoint inhibitors (ICI) (24).

Cases of OB are usually described in adult patients (median age is about 50 years old) and in the context of specific risk factors, such as following a lung transplant with allograft rejection, GVHD, connective tissue disease, and occupational exposure (25). Some cases have been recently reported after drug exposure to substances such as 5-fluorouracil, afatinib, rituximab, and ICIs (26), but only a few cases during MKI treatment (27, 28). It is also true that OB incidence has been increasing over time, as well as the list of OB-associated drugs (26). Here, we presented a case of OB in an elderly patient without any recognized risk factor. An association between OB and selpercatinib treatment in the current case could be raised because of the temporal association between OB feature onset and selpercatinib treatment initiation, and the absence of other risk factors potentially associated with OB.

Dynamic, ongoing pros and cons evaluation guided management choices in this case. It is known that the natural history of OB is highly inconstant, ranging from severe respiratory insufficiency over time to mild stabilized symptoms after initial impairment (20, 25). Treatment with corticosteroids, with or without immunosuppressive agents, has been pursued but with poor efficacy (25). Moreover, it is worth noting that in this case, clinical and imaging features were mild and fluctuating over time, without the impact of any specific therapy. On the other hand, selpercatinib has been shown to produce a significant increase in progression-free and treatment failure-free survival compared with standard treatments (i.e. cabozantinib or vandetanib) in patients with *RET*-mutant MTC (4). Accordingly, we pursued active surveillance of this AE, considering the interruption or dose reduction of selpercatinib only in case of relevant worsening of both clinical and imaging features that, indeed, have not happened, at least so far.

Additional diagnostic workup could be performed in cases of OB. Respiratory function tests, such as spirometry with Diffusion Lung Carbon Monoxide (DLCO), should be performed at OB diagnosis and during follow-up. Although an obstructive pattern is usually observed, cases without any defects, such as in our case, have also been reported (19, 20). Lung biopsy could be useful to demonstrate the histopathologic features of OB (20). In this case, it was avoided because of the highly suggestive clinical and imaging features for OB, as reported in other series (19), and the risk of major complications and fibrotic processes after biopsy that could even worsen the lung involvement (29, 30). Moreover, in OB cases, lung biopsy is suggested only if the diagnosis is still unclear after performing a CT scan and lung function tests. This is due to the variable distribution of lesions often leading to false negative tests and the invasive nature of this procedure (31).

In conclusion, we reported a clinical case of a patient treated with selpercatinib for advanced/progressive MTC with peculiar pulmonary toxicity characterized by highly suggestive fluctuating OB. To our knowledge, this is the first case reported, and for this reason, there are no established treatment guidelines. The lesson we learned from this case is that, as a general rule, AEs should be carefully managed by weighing the pros and cons of immediate therapy for the AE or a reduction/interruption of the anti-cancer therapy in favor of a more conservative approach. Therefore, if the patient remains asymptomatic, a careful follow-up and active surveillance of the AE is a viable option to consider, particularly for fluctuating OB.

## Declaration of interest

RE is a consultant for EISAI, Lilly, IPSEN, Bayer, and Roche; however, the description and management of this case were not influenced by this activity.

## Funding

This study has been supported by grants to R E from Associazione Italiana per la Ricerca sul Cancrohttp://dx.doi.org/10.13039/501100005010 (AIRC, Investigator grant 2018, project code 21790).

## Patient consent

Signed informed consent was obtained directly from the patient.

## Author contribution statement

All authors made individual contributions to authorship. CG, AP, RE, and AM were involved in the diagnosis and management of this patient and wrote the initial draft of the manuscript. CR, AC were involved in the imaging evaluation; CR, AC, and RE were involved in the management of the patient and revised the manuscript. All authors reviewed and approved the final draft.

## Data availability Statement

Data sharing is not applicable to this article, as no datasets were generated or analyzed during the current study.
